# Supplementary material for: Emergency department performance assessment using administrative data: A managerial framework
Source: PLoS One. 2023 Nov 2;18(11):e0293401. doi: 10.1371/journal.pone.0293401 (PMC10621983; doi:10.1371/journal.pone.0293401)
Supplement: S1 Table — (DOCX) [file pone.0293401.s001.docx]

**S1 Table 1. Experts’ general framework evaluation**

|  | **Controller** | **COU Clinical Director** | **ED Clinical Director** | **General Hospital Manager** |
| --- | --- | --- | --- | --- |
| Is, in your view, the collection of additional information required to enrich the framework? | N | Y | Y | Y |
| Is, in your view, the assignment of indicators to performance dimensions helpful in their interpretation? | Y | Y | Y | Y |
| Do you agree with the clustering of indicators in the presented performance dimensions? | Y | Y | Y | Y |
| Is, in your view, the framework comprehensive for multidimensional performance evaluation? | Y | Y | Y | Y |
| Is, in your view, the framework useful to support your professional activity? | Y | Y | Y | Y |
| Is, in your view, the framework easy to implement in practice? | N | Y | N | Y |
| Are you aware that administrative data can be used for performance assessment? | Y | Y | Y | N |
