# Supplementary material for: Emergency department performance assessment using administrative data: A managerial framework
Source: PLoS One. 2023 Nov 2;18(11):e0293401. doi: 10.1371/journal.pone.0293401 (PMC10621983; doi:10.1371/journal.pone.0293401)
Supplement: S2 Table — (DOCX) [file pone.0293401.s002.docx]

**S2 Table 1. Experts’ assessment of the framework’s content**

|  | **Controller** | | **COU Clinical Director** | | **ED Clinical Director** | | **General Hospital Manager** | |
| --- | --- | --- | --- | --- | --- | --- | --- | --- |
|  | ***M*** | ***D*** | ***M*** | ***D*** | ***M*** | ***D*** | ***M*** | ***D*** |
| How useful is, in your opinion, indicators’ group 1.1 (namely, *Temporal patient-related metrics* such as arrival to physician evaluation, bet-to-provider time, arrival to triage, etc.) for your personal system of incentives toward professional goals (M) and for the management of your operations (D)? 1: no useful at all / 5: very useful | 2 | 5 | 4 | 5 | 5 | 5 | 3 | 5 |
| How useful is, in your opinion, indicators’ group 2.1 (namely, *Temporal provider-related metrics* / Provider’s decision-related times such as arrival to decision to admit, admission order to ED departure, etc.) for your personal system of incentives toward professional goals (M) and for the management of your operations (D)? 1: no useful at all / 5: very useful | 2 | 5 | 5 | 5 | 5 | 5 | 3 | 5 |
| How useful is, in your opinion, indicators’ group 2.2 (namely, *Temporal provider-related metrics* / Intervention and procedural times such as time from triage to first analgesia, lab turnaround time, etc.) for your personal system of incentives toward professional goals (M) and for the management of your operations (D)? 1: no useful at all / 5: very useful | 2 | 4 | 5 | 5 | 5 | 5 | 3 | 5 |
| How useful is, in your opinion, indicators’ group 2.3 (namely, *Temporal provider-related metrics* / Triage time-efficiency such as begin triage to end triage time, triage presentation to registration complete time) for your personal system of incentives toward professional goals (M) and for the management of your operations (D)? 1: no useful at all / 5: very useful | 2 | 3 | 5 | 5 | 5 | 5 | 3 | 3 |
| How useful is, in your opinion, indicators’ group 3.1 (namely, *Quality patient-related metrics* / Patient leaves such as left against medical advice, left after medical screening exam rate, etc.) for your personal system of incentives toward professional goals (M) and for the management of your operations (D)? 1: no useful at all / 5: very useful | 3 | 5 | 4 | 3 | 3 | 3 | 1 | 1 |
| How useful is, in your opinion, indicators’ group 3.2 (namely, *Quality patient-related metrics* / Patient returns such as 72-hour return visit rate, unplanned return rate to ED of primary care provider, etc.) for your personal system of incentives toward professional goals (M) and for the management of your operations (D)? 1: no useful at all / 5: very useful | 2 | 3 | 5 | 5 | 3 | 3 | 5 | 5 |
| How useful is, in your opinion, indicators’ group 3.3 (namely, *Quality patient-related metrics* / Adverse events such as mortality/death in ED, rate of adverse events, etc.) for your personal system of incentives toward professional goals (M) and for the management of your operations (D)? 1: no useful at all / 5: very useful | 2 | 2 | 5 | 5 | 3.5 | 3.5 | 5 | 5 |
| How useful is, in your opinion, indicators’ group 4.1 (namely, *Quality provider-related metrics* / Treatment decisions / Procedures such as number CT scans, urinary catheter, etc.) for your personal system of incentives toward professional goals (M) and for the management of your operations (D)? 1: no useful at all / 5: very useful | 4 | 5 | 2 | 2 | 3 | 3 | 5 | 5 |
| How useful is, in your opinion, indicators’ group 4.2 (namely, *Quality provider-related metrics* / Treatment decisions / Drugs such as inappropriate antibiotic use, correct antibiotic use, etc.) for your personal system of incentives toward professional goals (M) and for the management of your operations (D)? 1: no useful at all / 5: very useful | 2 | 2 | 5 | 5 | 3 | 3 | 4 | 4 |
| How useful is, in your opinion, indicators’ group 4.3 (namely, *Quality provider-related metrics* / Treatment decisions / Examination thoroughness and results such as accuracy of ED diagnoses, incomplete vitals documented, etc.) for your personal system of incentives toward professional goals (M) and for the management of your operations (D)? 1: no useful at all / 5: very useful | 2 | 2 | 5 | 5 | 5 | 5 | 5 | 5 |
| How useful is, in your opinion, indicators’ group 4.4 (namely, *Quality provider-related metrics* / Treatment decisions / Guideline adherence such as adherence to clinical guidelines, compliance with evidence-based guidelines, etc.) for your personal system of incentives toward professional goals (M) and for the management of your operations (D)? 1: no useful at all / 5: very useful | 3 | 3 | 4 | 4 | 4 | 4 | 5 | 5 |
| How useful is, in your opinion, indicators’ group 5.1 (namely, *Throughput metrics* / Patient flow by disposition / Admission such as admission rate over percentage of patients admitted, patient rate admitted per morning / evening / night, etc.) for your personal system of incentives toward professional goals (M) and for the management of your operations (D)? 1: no useful at all / 5: very useful | 5 | 5 | 5 | 5 | 3 | 3 | 5 | 5 |
| How useful is, in your opinion, indicators’ group 5.2 (namely, *Throughput metrics* / Patient flow by disposition / Transfer such as number of transfers to and from ED, percentage of critically ill patients transferred, etc.) for your personal system of incentives toward professional goals (M) and for the management of your operations (D)? 1: no useful at all / 5: very useful | 4 | 4 | 3 | 3 | 4 | 4 | 5 | 5 |
| How useful is, in your opinion, indicators’ group 5.3 (namely, *Throughput metrics* / Patient flow by disposition / Discharge such as number of patients discharged per hour, discharge rate, etc.) for your personal system of incentives toward professional goals (M) and for the management of your operations (D)? 1: no useful at all / 5: very useful | 3 | 3 | 3 | 3 | 4 | 4 | 1 | 1 |
| How useful is, in your opinion, indicators’ group 5.4 (namely, *Throughput metrics* / Patient flow by disposition / Overall ED occupancy such as daily boarding hours, percentage patients treated, etc.) for your personal system of incentives toward professional goals (M) and for the management of your operations (D)? 1: no useful at all / 5: very useful | 3 | 3 | 5 | 5 | 4 | 4 | 5 | 5 |
