# Supplementary material for: Emergency department performance assessment using administrative data: A managerial framework
Source: PLoS One. 2023 Nov 2;18(11):e0293401. doi: 10.1371/journal.pone.0293401 (PMC10621983; doi:10.1371/journal.pone.0293401)
Supplement: Attachment — Submitted filename: Rebuttal letter.docx [file pone.0293401.s003.docx]

*Italic* font has been used for requests; a regular font for answers.

**Journal requirements**

*1. Please ensure that your manuscript meets PLOS ONE's style requirements, including those for file naming. The PLOS ONE style templates can be found at https://journals.plos.org/plosone/s/file?id=wjVg/PLOSOne_formatting_sample_main_body.pdf and*

*https://journals.plos.org/plosone/s/file?id=ba62/PLOSOne_formatting_sample_title_authors_affiliations.pdf*

We have changed the style of the manuscript according to the guidelines provided in the links above.

*2. You indicated that ethical approval was not necessary for your study. We understand that the framework for ethical oversight requirements for studies of this type may differ depending on the setting and we would appreciate some further clarification regarding your research.*

*Could you please provide further details on why your study is exempt from the need for approval and confirmation from your institutional review board or research ethics committee (e.g., in the form of a letter or email correspondence) that ethics review was not necessary for this study?*

*Please include a copy of the correspondence as an "Other" file.*

The study is theoretical, not practical. Actually, no administrative record related to any patient has been seen, nor cited, nor referred to in any way. However, a statement from our Research Ethics Committee has been attached. In the statement, the Committee has specified that the study poses no ethical concerns. Also a copy of their email has been added.

*3. Please provide additional details regarding participant consent. In the ethics statement in the Methods and online submission information, please ensure that you have specified (1) whether consent was informed and (2) what type you obtained (for instance, written or verbal, and if verbal, how it was documented and witnessed). If your study included minors, state whether you obtained consent from parents or guardians. If the need for consent was waived by the ethics committee, please include this information.*

*If you are reporting a retrospective study of medical records or archived samples, please ensure that you have discussed whether all data were fully anonymized before you accessed them and/or whether the IRB or ethics committee waived the requirement for informed consent. If patients provided informed written consent to have data from their medical records used in research, please include this information.*

The following statement has been added at the beginning of the Methods:

In this research, no administrative record related to any patient has been examined, cited, nor referred to. Data in this article consists of theoretical indicators extracted from published scientific papers (properly cited). On the other hand, for the interviews evaluating the framework, i.e., its indicators, doctors and managers provided their verbal consent. Anyway, the Ethical Committee expressed a favourable opinion on the diffusion of the contents in this manuscript.

…

The General Hospital Manager, the Controller, the ED Clinical Director and the COU (Complex Operating Unit) Clinical Director have been selected for the interviews. None of the interviewees mentioned anything that could be used to identify specific patients, clinical records, and/or the interviewees themselves. Interviews are therefore fully anonymous.

*4. In your Data Availability statement, you have not specified where the minimal data set underlying the results described in your manuscript can be found. PLOS defines a study's minimal data set as the underlying data used to reach the conclusions drawn in the manuscript and any additional data required to replicate the reported study findings in their entirety. All PLOS journals require that the minimal data set be made fully available. For more information about our data policy, please see http://journals.plos.org/plosone/s/data-availability.*

*Upon re-submitting your revised manuscript, please upload your study’s minimal underlying data set as either Supporting Information files or to a stable, public repository and include the relevant URLs, DOIs, or accession numbers within your revised cover letter. For a list of acceptable repositories, please see http://journals.plos.org/plosone/s/data-availability#loc-recommended-repositories. Any potentially identifying patient information must be fully anonymized.*

*Important: If there are ethical or legal restrictions to sharing your data publicly, please explain these restrictions in detail. Please see our guidelines for more information on what we consider unacceptable restrictions to publicly sharing data: http://journals.plos.org/plosone/s/data-availability#loc-unacceptable-data-access-restrictions. Note that it is not acceptable for the authors to be the sole named individuals responsible for ensuring data access.*

*We will update your Data Availability statement to reflect the information you provide in your cover letter.*

We have uploaded the supplementary materials (S1 and S2) and pointed to them in the text. The minimal data set consists of S1 and S2.

*5. Please include your full ethics statement in the ‘Methods’ section of your manuscript file. In your statement, please include the full name of the IRB or ethics committee who approved or waived your study, as well as whether or not you obtained informed written or verbal consent. If consent was waived for your study, please include this information in your statement as well.*

We have included the approval statement from our Ethical Committee in the methods. As specified above, we have received verbal consent from the interviewees, witnessed by three of the authors. The approval statement from the Ethical Committee has also been uploaded as a file ‘Other’.

*6. Please include captions for your Supporting Information files at the end of your manuscript, and update any in-text citations to match accordingly. Please see our Supporting Information guidelines for more information: http://journals.plos.org/plosone/s/supporting-information.*

We have included captions to the supplementary materials.

**Editor**

*Please just include ethical challenges regarding administrative data (example https://www.mdpi.com/2073-431X/12/6/114 ) and send to us back.*

We have included a citation to this work, which we find relevant in our field. Thanks for suggesting it.
